# Effect of Underwater Treadmill Gait Training With Water-Jet Resistance on Balance and Gait Ability in Patients With Chronic Stroke: A Randomized Controlled Pilot Trial

**DOI:** 10.3389/fneur.2019.01246

**Published:** 2020-02-12

**Authors:** Chae-gil Lim

**Affiliations:** Department of Physical Therapy, College of Health Science, Gachon University, Incheon, South Korea

**Keywords:** stroke, underwater treadmill gait training with water-jet resistance, balance, gait ability, hemiplegia

## Abstract

**Objective:** The purpose of this study was to determine the effects of underwater treadmill gait training with water-jet resistance and underwater treadmill gait training with ankle weights on balance and gait abilities in chronic stroke patients.

**Methods:** Twenty-two inpatients and outpatients with stroke-induced impairments were randomly assigned into two groups: an underwater treadmill gait training with water-jet resistance group (*n* = 11) and an underwater treadmill gait training with ankle weights group (*n* = 11). Participants received conventional physical therapy for 30 min and underwater treadmill gait training with water-jet resistance or ankle weights for 30 min. Intervention was performed 5 days a week for 4 weeks. The Balance System SD was used to assess static and dynamic balance. The GAITRite system was used to assess gait velocity, cadence, step length, stride length, and swing phase. All measurements were performed at the beginning of the study and 4 weeks after the intervention.

**Results:** The water-jet resistance group and ankle weights group showed significant improvement in static balance (*P* < 0.00 vs. *P* = 0.01), dynamic balance (*P* < 0.00 vs. *P* = 0.57), gait velocity (*P* < 0.00 vs. *P* = 0.037), cadence (*P* < 0.00 vs. *P* = 0.001), step length (*P* < 0.00 vs. *P* = 0.003), stride length (*P* < 0.00 vs. *P* = 0.023), and swing phase (*P* < 0.00 vs. *P* < 0.00). However, the static and dynamic balance ability score (*P* < 0.00), gait velocity (*P* < 0.00), cadence (*P* < 0.00), step length (*P* < 0.00), stride length (*P* < 0.00), and swing phase (*P* = 0.023) in the group that received underwater treadmill gait training with water-jet resistance improved more than in the group that received underwater treadmill gait training with ankle weights.

**Conclusions:** Our results demonstrated that underwater treadmill gait training with water-jet resistance is effective in improving static and dynamic balance as well as gait abilities in chronic stroke patients. Thus, training using underwater treadmill gait training with water-jet resistance may be useful in facilitating active rehabilitation in chronic stroke patients.

## Introduction

Stroke patients have impaired walking ability due to decreased balance and are prone to falls (1, 2). Falls cause injuries, resulting in decreased mobility, fear of falls, difficulty in resuming activities of daily living, and impaired balance and gait ability (3–6).

Weight-bearing treadmill training using a task-oriented approach is designed to improve balance and gait ability in hemiplegic patients (7–9). However, to provide treadmill training for stroke patients, it is necessary to promote a sense of stability. Therefore, a safety harness may be necessary for walking in water (10, 11).

Underwater treadmill training by using the buoyancy can be promoted to gait disturbance of stroke patients rather than land-based treadmill training because of effectively reducing body weight. In addition, water resistance can increase energy consumption by combining aerobic exercise and resistance exercise (12). Also, Underwater treadmill training reduces the burden on weight-bearing joints and the risk of falling, and provides resistance during movement in multiple directions (13). Thus, underwater aerobic exercise that reproduces the action of walking and running is increasingly popular (14).

Even when walking ability is restored in stroke patients, hip and knee joint flexion and ankle dorsiflexion are reduced (15). As a result, stroke patients have difficulty in dealing with obstacles more complicated than gait or stair climbing, and require proper flexion of the lower limb and dorsiflexion of the foot from the floor (16).

The authors previously reported that when a sandbag equivalent to 5% of body weight was worn on the affected ankle during weight-bearing treadmill training, the walking speed was improved, and the ratio of the swing phase was significantly increased in the hemiparetic lower limb and increased in the non-hemiparetic lower limb (17). A recent study reported that when weight is applied to the affected ankle during underwater walking, the support ratio increases and stability and symmetry increase (18). Underwater backward and forward treadmill walking against a variable-speed current reportedly led to increased step length and frequency (19).

Previous studies on treadmill training used resistance to improve walking ability by applying external weights to the lower extremities in chronic stroke patients. However, studies on underwater treadmill training in stroke patients using water-jet resistance or external weights only included a short training period or a cross-sectional study design.

This study compared the effect of water-jet resistance and ankle load training on balance and walking ability in stroke patients to identify intervention methods that can be applied in clinical practice.

## Methodology

### Setting, Study Design, and Participants

This study was a two-arm, parallel, and randomized controlled pilot trial with concealed allocation, and researcher and assistants blinding. All procedure of this study was approved by the Institutional Review Board of Gachon University (IRB No: 1044396-201612-HR-096-02) and registered at Clinical Research Information Service (CRiS), Republic of Korea (KCT0003576) and all participants signed an informed consent prior to beginning the study. In addition, this study conforms to all CONSORT guidelines as much as possible.

### Experimental Procedure

Patients with a history of stroke were recruited from a rehabilitation hospital in Incheon, Republic of Korea. Participants were enrolled in this study if 6 months or more had passed since onset of a unilateral hemispheric first stroke; the ability to walk at least 10 m was required (regardless of need for assistance), and the required mini-mental state examination (MMSE) score was at least 24. All patients were diagnosed with a chronic stroke as defined by computed tomography or magnetic resonance imaging. Patients with a cognitive, visual, or cardiorespiratory disorder (including cardiac pacemaker placement, heart failure, and arrhythmia), orthopedic intervention, hydrophobia, skin disease, and undergoing botulinum toxin injections within the prior year were excluded. Also, Patients with a pulse rate ≥100 beats per minute (bpm), a systolic blood pressure ≥180 mmHg, and a diastolic blood pressure ≥100 mmHg were excluded.

### Randomization and Masking

Twenty-two inpatients and outpatients with stroke-induced impairments were randomly assigned into two groups: an underwater treadmill gait training with water-jet resistance group (*n* = 11) and an underwater treadmill gait training with ankle weights group (*n* = 11). Randomization was intended to minimize an order effect. Baseline measurements of abilities were performed prior to randomization. Subsequently, each participant was allocated to one of the two groups via allocation codes included in consecutively numbered, sealed, opaque envelopes. Simple randomization was conducted using Microsoft Excel for Windows software (Microsoft Corporation, Redmond, WA, USA) by a researcher who was not involved in participant recruitment. To ensure masking, protocols and intervention order were not revealed to participants or clinical evaluators.

### Interventions Procedures

Hydrotherapy protocols included shallow water flowing in various combinations of buoyancy, hydrostatic pressure, turbulence, and resistance (depending on water level and treadmill speed). A higher water levels provide higher buoyancy and hydrostatic pressure, but higher velocities produce more turbulence and resistance (20). The slope of the underwater treadmill was horizontal, and the height of the water was the height of the xiphoid process (21). The temperature of water was 34°C, which is suitable for functional training while minimizing the physiological changes and stabilizing the deep body temperature (18). The treatment room temperature was set at 26°C to reduce the difference between room temperature and water temperature (22).

**Figure 1:**
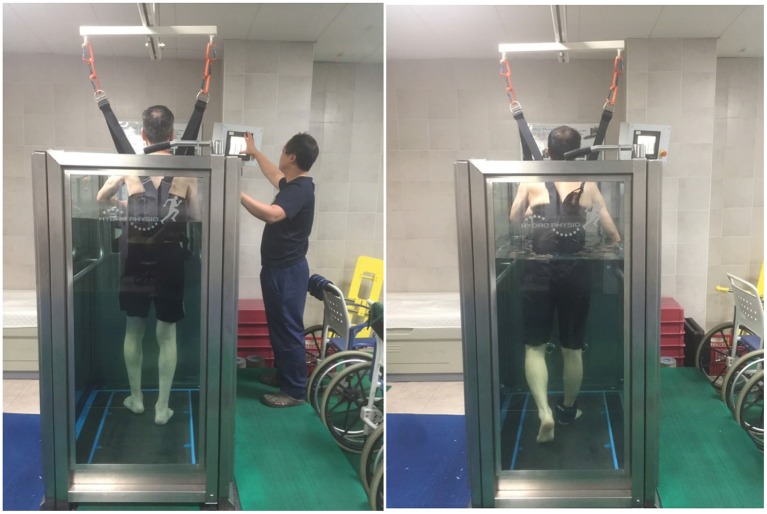
Underwater treadmill training with water-jet resistance **(left)** and Underwater treadmill training with ankle weight **(right)**.

Participants received a conventional physical therapy program for 30 min and gait training on an underwater treadmill (Focus, Hydro Physio, Nottingham, Nottinghamshire, UK), with water-jet resistance at 442 L/min against the anterior the shin, or wore an ankle weight equivalent to 5% of body weight for 30 min (Figure 1). The initial speed of this program based on the study that treadmill training performed at the fastest speed that stroke patients can perform was significantly increased in stride length, walking speed, and step length than that of fixed speed treadmill training. The training was performed at the maximum speed that the patients could do within the range of the walking speed was 1–4 m/s. At this time, when the patient was breathing or experiencing difficulty during training, the maximum speed was reduced by one step (23). We applied one of these methods to the patients considering of their ability. In all interventions and assessments, if the patient complained of discomfort, immediately stop and take a rest. The intervention was performed 5 days a week for 4 weeks (17).

### Outcome Measures

The general characteristics were collected through file audit and self-report. The primary outcomes were the Static and dynamic balance abilities by measured with the Balance System SD (Biodex Medical Systems, Inc., Shirley, NY, USA). The secondary outcomes were the changes in gait abilities. The GAITRite system (CIR Systems, Inc., PA, USA) was used to assess gait velocity, cadence, step length, stride length, and swing phase. All measurements were performed at the beginning of the study and 4 weeks after the intervention.

### Sample Size Estimation

We estimated a minimum acceptable sample size of 21 patients per group to achieve a power of 0.8 with a significance level (α) of 0.05 using a 1-sided, 2-sample *t*-test (G^*^Power 3.1) but we realistic enrolled 30 patient and was referenced that 21 patients would be necessary based on an inter-groups difference in endurance training with hydrotherapy in a previous trial (12).

### Data Analysis

Statistical analyses were performed using SPSS for Windows Version 18.0 (IBM, Armonk, NY, USA). The Kolmogorov–Smirnov test was used to determine the normality of parameter distributions. For a normal distribution, continuous data were expressed as the mean ± standard deviation, and as a percentage for categorical data; parametric tests such as an independent-samples *t*-test or the χ^2^ test were used to compare baseline characteristics of the two groups. A paired *t*-test was used for within-group comparisons, and an independent *t*-test was used for between-group comparisons. The level of significance was set at α = 0.05.

## Results

**Figure 2:**
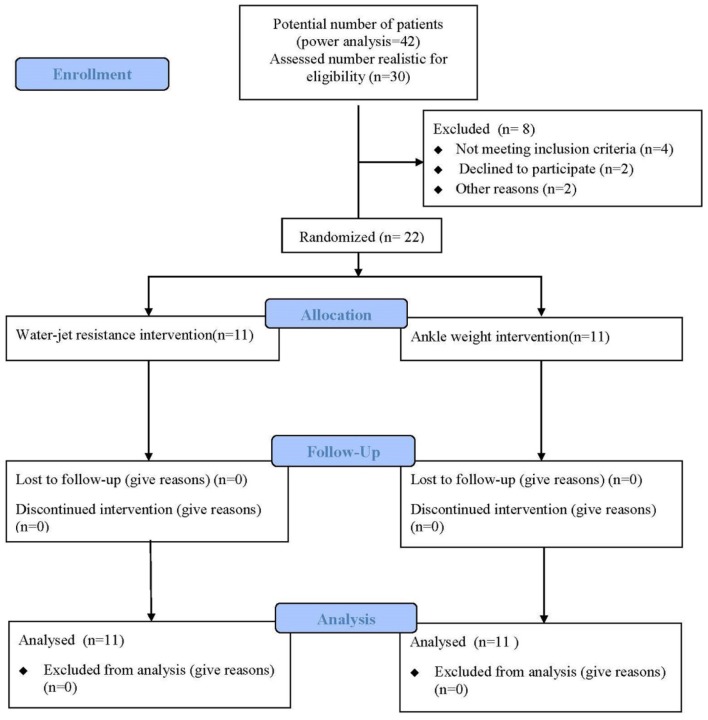
Flow diagram of this study. Twenty-two individuals were enrolled in the study and were randomly assigned to the water-jet group (*n* = 11) or the ankle weight group (*n* = 11).

Between Jun 2017 and Dec 2017, a total of 30 patients were admitted to the rehabilitation center, 22 fulfilled the inclusion criteria. Participants were randomly assigned to an underwater treadmill gait training with water-jet resistance group (*n* = 11) or an underwater treadmill gait training with ankle weights group (*n* = 11). All 22 participants completed the study (Figure 2). General baseline characteristics are shown in Table 1. Recorded characteristics included gender, age, height, weight, lesion side, post-stroke duration, and MMSE scores. The mean ± SD age of the patients was 51.90 ± 9.62 years, and post-stroke duration was 9.63 ± 2.61 months. Baseline demographic characteristics such as gender (males/females, 7/4 vs. 9/2), age (54.63 ± 7.25 vs. 49.18 ± 12.00 years), lesion side (right/left, 5/6 vs. 3/8), and post stroke-duration (10.18 ± 2.92 vs. 9.09 ± 2.30 months), and MMSE scores (25.63 ± 1.43 vs. 25.81 ± 1.66 score) were not significantly different between to an underwater treadmill gait training with water-jet resistance group and an underwater treadmill gait training with ankle weights group (*P* > 0.05).

**Table 1:** General characteristics of the two groups by randomization assignment.

	**Water jet group (*n* = 11)**	**Ankle weight group (*n* = 11)**	***P***
Gender (male/female)	7:4	9:2	0.34^a^
Age (years)	54.63 ± 7.25	49.18 ± 12.00	0.21^b^
Height (cm)	165.36 ± 5.33	172.18 ± 10.39	0.07^b^
Weight (kg)	66.45 ± 7.35	71.72 ± 12.73	0.25^b^
Lesion side (right/left)	5:6	3:8	0.38^a^
Post-stroke duration (month)	10.18 ± 2.92	9.09 ± 2.30	0.34^b^
MMSE^*^(score)	25.63 ± 1.43	25.81 ± 1.66	0.79^b^

*Data are expressed as mean ± SD or n (%)*.

* *MMSE, mini mental state examination*.

a *The P-value was obtained using a χ^2^*.

b *The P-value was obtained using an independent t-tests*.

Both group showed significantly improved static balance. In the water-jet group, the static balance score improved from 1.16 ± 0.32 to 0.49 ± 0.17 score (*P* < 0.00) and the dynamic balance score improved from 3.57 ± 1.45 to 1.78 ± 0.88 score (*P* < 0.00). In the ankle weight group, the static balance score improved from 1.10 ± 0.42 to 0.95 ± 0.32 score (*P* = 0.01) and the dynamic balance score little improved from 3.15 ± 0.80 to 3.09 ± 0.91 score (*P* = 0.57). However, the change in the static balance ability score in the water- jet group improved more than in the ankle weight group (*P* = 0.00; effect size = 0.73), and the change in the dynamic balance ability score in the water-jet group improved more than in the ankle weight group (*P* = 0.00; effect size = 0.76; Table 2).

**Table 2:** Changes in static and dynamic balance within each group and between the two groups.

			**Intragroup**		
			**Water jet group (*n* = 11)**	**Ankle weight group (*n* = 11)**	**Intergroup (*p*)**
Balance (score)	Static	Pre	1.16 ± 0.32	1.10 ± 0.42	
		Post	0.49 ± 0.17	0.95 ± 0.32	
		*P*	0.00^a^	0.01^a^	
		Post-Pre	−0.67 ± 0.31	−0.15 ± 0.16	0.000^b^
	Dynamic	Pre	3.57 ± 1.45	3.15 ± 0.80	
		Post	1.78 ± 0.88	3.09 ± 0.91	
		*P*	0.00^a^	0.57	
		Post-Pre	−1.79 ± 1.00	−0.06 ± 0.00	0.000^b^

*Data are presented as mean ± SD*.

a *P <0.05. The P-value was obtained using a paired t-test*.

b *P <0.05. The P-value was obtained using an independent t-test*.

In temporal parameter of gait ability, the gait velocity improved (*P* < 0.00 vs. *P* = 0.037) and the spatial parameters as cadence (*P* < 0.00 vs. *P* = 0.001), step length (*P* < 0.00 vs. *P* = 0.003), stride length (*P* < 0.00 vs. *P* = 0.023), and swing phase (*P* < 0.00 vs. *P* < 0.00) values in both groups were increased compared to pre-intervention values. However, the velocity (*P* = 0.00; effect size = 0.87), cadence (*P* = 0.00; effect size = 0.91), step length (*P* = 0.00; effect size = 0.88), stride length (*P* = 0.00; effect size = 0.95), and swing phase (*P* = 0.023; effect size = 0.54) values in the water jet group improved more than in the ankle weight group (Table 3).

**Table 3:** Changes in gait ability within each group and between the two groups.

		**Intragroup**		
**Gait ability**		**Water jet group (*n* = 11)**	**Ankle weight group (*n* = 11)**	**Intergroup (*p*)**
Velocity (cm/s)	Pre	54.34 ± 19.43	55.01 ± 17.56	
	Post	76.40 ± 18.95	57.85 ± 18.16	
	*P*	0.000^a^	0.037^a^	
	Post-Pre	22.06 ± 6.69	2.60 ± 1.76	0.000^b^
Cadence (steps/m)	Pre	70.73 ± 15.00	73.89 ± 10.85	
	Post	88.52 ± 16.75	76.49 ± 11.56	
	*P*	0.000^a^	0.001^a^	
	Post-Pre	17.79 ± 6.38	2.60 ± 1.76	0.000^b^
Step length (cm)	Pre	38.40 ± 9.48	41.07 ± 10.62	
	Post	54.36 ± 7.65	43.56 ± 11.14	
	*P*	0.000^a^	0.003^a^	
	Post-Pre	15.96 ± 4.81	2.48 ± 2.16	0.000^b^
Stride length (cm)	Pre	63.21 ± 12.89	68.36 ± 9.93	
	Post	82.43 ± 12.37	70.09 ± 9.72	
	*P*	0.000^a^	0.023^a^	
	Post-Pre	15.96 ± 4.81	2.48 ± 2.16	0.000^b^
Swing phase (%)	Pre	32.78 ± 4.56	33.23 ± 1.93	
	Post	39.39 ± 2.21	36.95 ± 2.08	
	*P*	0.000^a^	0.000^a^	
	Post-Pre	6.60 ± 3.50	3.71 ± 1.04	0.023^b^

*Data are presented as mean ± SD*.

a P <0.05. The P-value was obtained using a paired t-test.

b *P <0.05. The P-value was obtained using an independent t-test*.

## Discussion

The results demonstrate that a 4 week underwater treadmill gait training program with water-jet resistance had a beneficial effect on static and dynamic balance ability scores, gait velocity, step length, stride length, and swing phase in patients with chronic stroke when compared with patients who underwater treadmill gait training with ankle weights. These results are of special interest because little evidence is available on the effect of aquatic intervention on balance and gait ability in stroke patients.

The typical gait pattern in stroke patients is characterized by unstable weight shifting to the affected side during walking, resulting in asymmetric and slow gait. In a study comparing postures in stroke patients and the elderly, it was reported that the stroke patients had an increased risk of falling because they exhibited asymmetric posture and greater postural sway than healthy elderly subjects (24). Wing et al. (25) suggested that whole-body intensive rehabilitation (3–6 h/day, 4–5 days/week, for ≥2 weeks) is effective for improving postural balance and functional mobility in stroke patients. To address these findings, water has been used in rehabilitation. Water provides a safe environment for patients by reducing the risk of falls. In addition, warm water can exert a therapeutic effect by alleviating pain or spasticity. Warmth can increase skin temperature, dilate peripheral blood vessels, increase blood supply, accelerate muscle relaxation, alleviate pain or muscle cramps, and improve balance (26).

Berger et al. (27) reported that 6 weeks of aquatic therapy improved postural control. Zhu et al. (28) suggested that a relatively short program (4 weeks) of hydrotherapy exercise resulted in a large improvement in a small group (*n* = 14) of individuals with relatively significant balance and walking dysfunction following a stroke. Mentiplay et al. (29) demonstrated that the strength of the knee extensors shows a poor-to-moderate correlation with gait velocity. Another study (30) determined that the static strength of lower limb muscles on the paretic side was correlated significantly with gait velocity and cadence in stroke patients. In agreement with a previous study, we found that balance and gait ability improved in chronic stroke patients. Improvements in knee range of motion resulted from repetitive movement in the sagittal plane by the anterior of the shin against water-jet resistance.

In contrast to these studies, Lee et al. (31) reported that a 4 week aquatic treadmill exercise program had a beneficial effect on muscle strength but not motor function and balance in subacute stroke patients, possibly because improvements were observed in all groups with conventional rehabilitation therapy in the subacute phase. In contrast, our study added water-jet resistance and participants had chronic strokes.

We also examined the effects of water-jet resistance on gait abilities. Previous reports showed that treadmill training may be more effective than conventional gait training in improving gait parameters such as functional ambulation, stride length, percentage of paretic single stance period, and gastrocnemius muscular activity (7). A recent systematic review reported that aquatic therapy improves dynamic balance and gait performance in individuals with neurological disorders, especially those with multiple sclerosis, Parkinson's disease, and stroke (32). We found that although velocity, cadence, step length, stride length, and swing phase in both groups tended to improve after 4 weeks of aquatic therapy, the difference in cadence between groups was insignificant. These results reflect the fact that water-jet resistance was applied against all areas below the knee, but that ankle weights seemed to play a role in limiting the effect of other areas and prevented ankle floating.

In our study, we had clinically significant effect sizes and high power for detecting statistically significant changes in the velocity (effect size = 0.87), cadence (effect size = 0.91), step length (effect size = 0.88), stride length (effect size = 0.95), and swing phase (effect size = 0.54).

This study had several limitations. First, the water jet resistance and ankle weights were applied at different locations (anterior of shin and ankle) and the same load (442 L/min) was applied in all subjects, regardless of sex, age, height, and weight. Although various levels of difficulty were provided according to the general characteristics of the subjects and study parameters were changed according to individual balance and gait ability, there was a limit in providing diversity. This led to some difficulty in adapting training for each subject, using the same applications. Therefore, further studies will need to address these limitations. Second, the number of subjects was small, and the subjects were limited to those with chronic stroke, which limited the ability to generalize the results of underwater treadmill gait training with water-jet resistance. Finally, we could not test lower limb strength. Based on these limitations, a more detailed training method should be used to identify the effects of this study.

## Conclusion

The present study showed that a 4 week underwater treadmill gait training program with water-jet resistance had a beneficial effect on static and dynamic balance ability scores, gait velocity, cadence, step length, stride length, and swing phase in patients with chronic stroke when compared with patients who underwater treadmill gait training with ankle weights. Future studies should assess results in a larger cohort of subjects with various durations since stroke onset.

## Data Availability Statement

The data used to support the findings of this study are available from the corresponding author upon request.

## Ethics Statement

All procedure of this study was approved by the Institutional Review Board of Gachon University (IRB No: 1044396-201612-HR-096-02).

## Author Contributions

CL makes substantial contributions to conception and design, acquisition of data (two assistant researcher help), data analyzing, substantial contributions to interpreting data, drafting the article and revising it critically for important intellectual content, and final approval of the version to be submitted.

### Conflict of Interest

The author declares that the research was conducted in the absence of any commercial or financial relationships that could be construed as a potential conflict of interest.
